# Role of genomics literacy in reducing the burden of common genetic diseases in Africa

**DOI:** 10.1002/mgg3.776

**Published:** 2019-05-26

**Authors:** Gerald Mboowa, Ivan Sserwadda

**Affiliations:** ^1^ Department of Immunology and Molecular Biology College of Health Sciences, Makerere University Kampala Uganda; ^2^ Department of Medical Microbiology College of Health Sciences, Makerere University Kampala Uganda

**Keywords:** Africa, education, genetics, genomics, literacy

## Abstract

**Background:**

In Africa, health practitioners and the current knowledge of the public on genetics and genomics is still very low and yet this has potential to reduce the burden of common genetic diseases. Many initiatives have promoted genomic research, infrastructure, and capacity building in Africa. What remains to be done is to improve genomics literacy among populations and communities while utilizing an array of strategies. Genomic literacy and awareness are key in the management of genetic diseases which includes diagnosis, prevention of complications and therapy. Africa is characterized by great cultural and language diversity thereby requiring a multidisciplinary approach to improving public and community genomics literacy and engagement. However, this is further complicated by having the fact that sub‐Saharan Africa is comprised of countries with the lowest literacy rates in the world.

**Methods:**

We applied the Preferred Reporting Items for Systematic Reviews and Meta‐Analyses guidelines to review genomic literacy in Africa using PubMed database.

**Results:**

We found very limited evidence of genomics literacy for genetic diseases in Africa.

**Conclusion:**

We propose a number of approaches that if adopted will significantly increase the genomic literacy and reduce the burden of genetic diseases in Africa.

## 1 INTRODUCTION

It has been estimated that more than 7,000,000 babies are born each year with either a congenital abnormality or a genetic disease, and that up to 90% of the births occur in low‐ or middle‐income countries (Christianson, Howson, & Modell, 2005). A minimum estimate suggests that an excess of 300,000 children are born each year with either sickle cell anemia (SCA, OMIM 603903) or one of its variants or a form of thalassemia (Modell & Darlison, 2008). In 2012, the cost of hospitalization of a child with SCA in Nigeria was estimated at $24,278.37 (Adegoke, Abioye‐Kuteyi, & Orji, 2014). Scientists in sub‐Saharan Africa (SSA) have had a real opportunity to break through and join their colleagues on the world stage in pushing forward the frontiers of research in genomics (Bekele et al., 2014) however, much remains to be done in improving public genomics literacy.

Given the importance of Africa to studies of human origins and disease susceptibility (Gurdasani et al., 2015), there is exponential growth in the interest and implementation of genomics research in Africa which has been facilitated by the Human Hereditary and Health in Africa (H3Africa) initiative. The aim of H3Africa is to promote a contemporary research approach to the study of genomics and environmental determinants of common diseases in African populations (Adebamowo et al., 2018). Health care benefits greatly from the unprecedented and ongoing work elucidating the genetic/genomic basis of health, illness, disease risk, and treatment response (Calzone et al., 2010). The progress in genetics and genomics is applicable to the entire spectrum of healthcare, all health professionals (Calzone et al., 2010), communities and the general public. In Africa, approaches such as genomics literacy and awareness for the communities and healthcare workers are key in the management of genetic diseases which includes diagnosis, prevention of complications and therapy. However, the healthcare workers and the populations with which they work are not well‐informed about the role of genomics in health and disease states. The shortage of healthcare workers in SSA continues to be a pressing reality. The healthcare practices and current educational curricula at all levels in African institutions have not adequately adopted genomic literacy (Wonkam, Njamnshi, & Angwafo, 2006) and yet this offers opportunities that can greatly change the distribution of common genetic diseases on the continent. Analysis of in‐depth interviews has already suggested that genomics medicine may have an impact on disease surveillance, diagnosis, treatment and prevention in Africa (Munung, Mayosi, & de Vries, 2018).

## 2 METHOD

**Figure 1:**
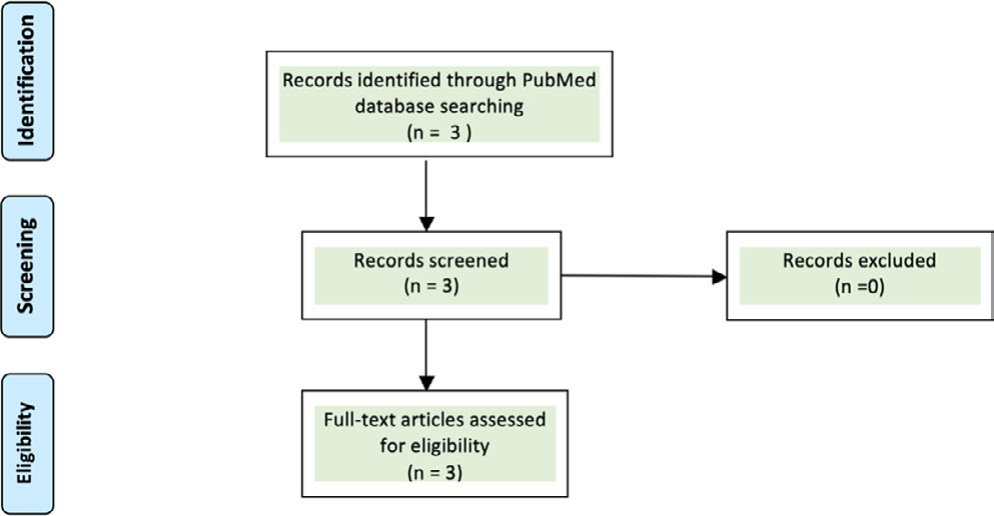
PRISMA (Preferred Reporting Items for Systematic Reviews and Meta‐Analyses) Workflow

We carried out a PubMed literature search using PRISMA (Preferred Reporting Items for Systematic Reviews and Meta‐analyses), a tool developed for the reporting of systematic reviews and meta‐analyses (Moher, Liberati, Tetzlaff, & Altman, 2009). Our search strategy included ("genomics"[MeSH Terms] OR "genomics"[All Fields]) AND ("education"[Subheading] OR "literacy"[All Fields] OR "literacy status"[MeSH Terms] OR ("literacy "[All Fields] AND "status"[All Fields]) OR " literacy status"[All Fields] OR "literacy"[All Fields] OR " literacy "[MeSH Terms]) AND ("genetic diseases, inborn"[MeSH Terms]) OR ("genetic"[All Fields] AND "diseases"[All Fields] AND "inborn"[All Fields]) OR "inborn genetic diseases"[All Fields] OR ("genetic"[All Fields] AND "diseases"[All Fields]) OR "genetic diseases"[All Fields]) AND ("africa"[MeSH Terms] OR "africa" AND "community" OR "outreach") as keywords and found very limited evidence of genomics literacy for genetic diseases in Africa as seen in Figure 1. In this review, we summarize the approaches that can reduce the burden of genetic diseases in Africa. We propose that the above aim can be achieved through the following strides; medical curricula revision, introduction of new graduate programs in genetics and genomics, workshops and fellowships, massive open online courses (MOOCS), community outreaches and genomic counseling amongst others.

## 3 RESULTS

Our PRISMA PubMed search returned only a count of 3 publications. Table 1 summarizes these articles.

**Table 1:** Summary of the PRISMA PubMed search

Reference	Title of the publication	Publication summary/conclusion
De Vries et al. (2011)	Ethical issues in human genomics research in developing countries	Many ethical issues are raised when genomics research is conducted on populations that are characterized by lower average income and literacy levels, such as the populations included in MalariaGEN. It is important that such issues are appropriately addressed in such research. Our experience suggests that the ethical issues in genomics research can best be identified, analysed and addressed where ethics is embedded in the design and implementation of such research projects
Wonkam, Muna, Ramesar, Rotimi, and Newport (2010)	Capacity‐building in human genetics for developing countries: initiatives and perspectives in sub‐Saharan Africa	Governments and international health agencies need to recognise that genetics is important to the global medical community. The initiatives of African geneticists need advocacy and encouragement from the international community
Burnham‐Marusich et al. (2016)	Prevalence of Sickle Cell Trait and Reliability of Self‐Reported Status among Expectant Parents in Nigeria: Implications for Targeted Newborn Screening	Low numbers of accurate parental self‐reports, coupled with a high SCT prevalence in Nigeria, could limit the efficacy of targeted newborn screening. However, our data indicate that it is feasible to integrate sickle cell screening for pregnant women with existing, community‐based health care programs developed by the President's Emergency Plan for AIDS Relief (PEPFAR), such as the HBI. Expanding screening programs could enable the development of targeted newborn screening based on maternal genotype that could identify all newborns with SCD in resource‐limited settings

Abbreviation: SCD, sickle cell disease.

### 3.1 Revision of medical curricula to include genomics modules

In the postgenomic era, bioinformatics analysis skills have become an all but unavoidable need for life scientists (Atwood et al., 2015; Madlung, 2018). However, integration of genomics modules into the undergraduate medical curricula remains unrealized, in part due to a lack of instructors. This applies to undergraduate medical courses in medical schools. Most healthcare workers in current practice have not had genomics literacy during their training. This is true for even some countries in the developed world where most medical practitioners in primary health care have not had a single hour of instruction in genetics as part of their formal training (Calzone et al., 2010) within their colleges or universities. Resources for clinicians and scientists to study the relationship among diseases, genes and variants continue to be developed (Popejoy et al., 2018) and this requires that medical training must revise their curricula to ensure that the current medical graduates are well‐versed in the genomic era. Revision of curricula can be extended to tertiary institutions that train allied health professionals. However, even school teachers should be involved in efforts that improve genomic literacy in the schools.

### 3.2 Introduction of new graduate programs in genetics and genomics

Genomics and bioinformatics approaches aiming to improve human health are revolutionizing medicine (Mulder, Adebiyi, et al., 2016). The continent of 55 countries, there less than 10 universities that have either undergraduate or graduate programs in genetics and genomics and of which more than half are found in South Africa. Bioinformaticists are in short supply everywhere (Pennisi, 2011) as are genomicists, but a number of universities in Africa have started training programs in genomics and bioinformatics. Currently, the use of genomics in the clinical settings in Africa is dominated by the genotyping of single genes with the aim of elucidating genetic etiologies for single‐gene disorders such as SCA and Downs syndrome (Modell & Darlison, 2008; Mulder, Adebiyi, et al., 2016). Even so, this service is still largely unavailable in many healthcare settings in Africa.

With support from NIH Common Fund, National Human Genome Research Institute and Fogarty to H3Africa Global Health Bioinformatics Research Training Program, a total of four groups in SSA have been funded to develop sustainable training programs to address the need for genomics research expertise in the H3Africa Consortium. The introduction of a cost‐effective Illumina H3Africa Consortium 2.5M African‐specific genome‐wide association study genotyping chip will certainly improve genomic research among African populations by African‐based researchers making these graduate training programs essential in this facet. The African Genome Variation Project provides a resource with which to design, implement and interpret genomic studies in SSA and worldwide (Gurdasani et al., 2015). An essential pre‐requisite for studies on human genetics is accurate, reliable (Butali et al., 2015), and standard phenotyping. Phenotypes are sets of observable characteristics and they are the product of the interaction between genotypes and the environment/disease outcome. A critical number of competent trained professionals will be necessary to sustain the achievements in genomic investments made on the continent. These will be involved at all levels including public engagement on genomics issues, policy formulations, healthcare provision, and ethics in carrying out genomic research.

Clinical genetics as a specialty should be introduced at medical schools across Africa. It must be unique and tailored towards disease conditions that are relevant in the African settings and populations. An estimated 303,000 newborns die within 4 weeks of birth every year, worldwide, due to congenital anomalies and may be much higher some less developed parts of the world. Most infants with a severe congenital disorder such as; hemoglobinopathies (OMIM 613985), Down syndrome (OMIM 190685), congenital heart disease, malformation of the urinary tract, and cystic fibrosis (OMIM 219700) are particularly susceptible to infections, and in many parts of the world, the affected children simply vanish within the general infant and childhood mortality without attracting any special notice. This is largely attributed to lack of the clinical geneticists compared to the patients in most in most African countries. Moreover, this fact is even present in the developed world such as in the United States which has a ratio five clinical geneticists per million populations (Zhang & Li, 2014). In many African institutions and hospitals, departments such as Genetics, Molecular genetics, Clinical genetics, Medical genetics or Cytogenetics are rare largely due to lack of this human resource and appropriate infrastructure. South Africa has the best trained medical geneticists, genetic counselors and medical scientists and genetic services in Africa (Kromberg, Sizer, & Christianson, 2013) while other countries are yet to appreciate the genetic services in healthcare sector. In Rwanda, the department of Medical Genetics at the faculty of medicine at the National University of Rwanda was opened in 2006 and has since provided genetic services to patients from within Rwanda and the surrounding regions (Mutesa et al., 2010; Uwineza & Mutesa, 2015).

### 3.3 Genomics workshops, fellowships and MOOCS for the healthcare workers

Human genetics and genomics fellowships are very important to the African faculty intending to introduce and train students of graduate genomics programs. This is important because they have had no undergraduate programs of genomics or even courses in their prior formal training. Many Africans from local institutions have been offered genomics fellowship opportunities at universities in Europe and America with the hope that they return to their institutions to engage in knowledge and skill transfer (Mlotshwa et al., 2017). These fellowships range from short to long‐term. In cases where trainees have returned to their home institutions, this has impacted the genomics capacity building and human resource, both fundamental and critical requirements for sustainable development (Munung, Mayosi, & de Vries, 2017).

In the past, a number of genomics workshops have been carried out on the continent by groups such as H3ABioNet (https://h3abionet.org/) that runs a number of specialized genomics courses, the African Biosciences Limited—the Basic Genomics and Bioinformatics Training Workshop (https://africanbio.com/event), the African Centre of Excellence for Genomics of Infectious Diseases—the foundational training workshop in Molecular biology and genomics (http://acegid.org/) and Wellcome Trust Advanced Courses—Genomic Epidemiology in Africa (https://www.malariagen.net/network/capacity-building/courses-and-workshops).

With changing student lifestyles and fast‐developing technology, universities are increasingly offering more “flexible” learning environments. A few online courses have been developed and successfully carried out in Africa to improve genomic medicine. MOOCs have received wide subscription all over the world and in Africa a few initiatives such as; the African Genomic Medicine Training Initiative, a MOOC designed and developed to train genomic medicine for African‐based healthcare professionals with the purpose of improving genetics and genomics knowledge, attitudes and skills. This course was first carried out as, "Introduction to genomic medicine for nurses in Africa". Nurses constitute 45–60 percent of the entire health workforce and are responsible for a broad range of services (Dovlo, 2007). Nurses have intimate knowledge of the patient's, family's, public's trust, and community's perspectives (Calzone et al., 2010). If well furnished with knowledge of role of genetics/genomics in health and disease, they would most importantly improve genomic literacy and education as well as engagement in communities where they work. The second is the 3 months’ online course developed by H3ABioNet's called "Introduction to Bioinformatics (IBT)" (Gurwitz et al., 2017) in which genomics modules are taught to participants from various backgrounds, but majority are students at African universities and other institutions. Other platforms that offer free online genomics MOOCs include the "FutureLearn", which is a United Kingdom based digital literacy platform that periodically offers courses such as “The Genomics Era: the Future of Genetics in Medicine”, Clinical Bioinformatics: Unlocking Genomics in Healthcare (https://www.futurelearn.com/courses/bioinformatics), Using Personalized Medicine and Pharmacogenetics (https://www.futurelearn.com/courses/personalized-medicine), Genomic Technologies in Clinical Diagnostics: Next Generation Sequencing (https://www.futurelearn.com/courses/next-generation-sequencing), Whole Genome Sequencing: Decoding the Language of Life and Health (https://www.futurelearn.com/courses/whole-genome-sequencing), Genomic Technologies in Clinical Diagnostics: Molecular Techniques, and Genomic Medicine: Transforming Patient Care in Diabetes (https://www.futurelearn.com/courses/diabetes-genomic-medicine) amongst others.

Since genomics is relatively new in Africa, many practicing healthcare professionals have a limited understanding of the role of genetics and genomics in diseases and health. To overcome this challenge, hospitals and health systems should introduce Genomics Continuing Medical Education Opportunities. The mode of delivery and the content can range from basics to more specialized content depending on the qualifications of the participants. Health systems planners and administrators should adopt and encourage partnerships with institutions that can extend genomics workshops, fellowships and MOOCS for their healthcare workers.

### 3.4 Community outreach strategies

Community engagement enhances a community's ability to address its own health needs and health disparities issues while ensuring that instructors understand community priorities (Ahmed & Palermo, 2010). However, this requires instructors with good understanding of genomics and its role in health and disease as well as experience with effective methods of engaging communities.

In most circumstances, people turn to practitioners for topic‐specific expertise when questions arise or help is needed (Scheitle, Johnson, & Ecklund, 20188). For this reason, individuals who have genomic expertise can be asked to deliver expert talks and public engagement to improve the level of knowledge, beliefs, myths, and attitudes about the risk factors for genetic diseases in communities. A variety of platforms can be utilized such as; print media (magazines and new papers), radio/television talk shows, genomics seminars at universities/tertiary institutions as well as secondary and primary school, genomics comic books (Mboowa et al., 2018), blogs and a range of social media platforms. The media plays a great role in disseminating scientific messages and in propagating or reducing misconceptions to the public (Hurle et al., 2013). Therefore, if well utilized, it can be very important in improving genomic literacy in communities.

Community and public outreach and engagement in genomics in Africa is still a complex issue largely because scientists have not yet been able to consider public engagement as an important aspect of their responsibility. Different strategies are required to enable communities understand the role of genetics in health and diseases. Many of the genomic technical terms require translation into common usage before engaging the public and non‐scientific communities. Africa is a continent with the highest linguistic diversity estimated between 1,000 and 2,500 languages (Ouane & Glanz, 2010), yet choosing to translate materials only into the most commonly spoken languages would raise ethical concerns.

In Africa, religious and cultural leaders have a lot of influence on society (Manguvo & Mafuvadze, 2015). If they are equipped with the right message they would be capable of improving genomic literacy. A classic example would be in the case where traditional marriages may require individuals to present evidence of sickle cell carrier status screening prior to marriage. This coupled with counseling would see a reduction in the sickle cell disease. Such a similar approach saw these leaders taking a pivotal role in the fight against HIV infections by ensuring mandatory pre‐marital HIV testing for all intending couples in many societies in Africa (Umar & Oche, 2012). Therefore, a combination of genetic testing for common diseases and increased genetic literacy amongst influential leaders in the communities would result into a reduction in the burden of these diseases.

Other ways to increase community engagement and awareness include encouraging discussions, target and train local leaders on ground who form part of health teams and local authorities. It is crucial to equally identify and train other stakeholders such as policymakers and legislatures at different levels in communities. These act as “genomics ambassadors” enabling appropriate policies to be enacted.

### 3.5 Genomic counseling services adopted by national healthcare systems to offer guidance on both rare and common genetic disorders to communities

Genetic counseling within high‐risk populations has greatly reduced the burden of genetic diseases. Sickle cell disease (SCD, OMIM603903) is one of the most common genetic causes of illness and death in the world (Makani, Ofori‐Acquah, Nnodu, Wonkam, & Ohene‐Frempong, 2013) and up to 75% of SCD in newborns world‐wide occurs in SSA (Mulder, Nembaware, et al., 2016). SCD is a heterogeneous disorder with a highly variable clinical spectrum (van der Land et al., 2013) and its genetic haplotypes are defined by restriction of fragment length polymorphisms in the *β*‐globin locus (Makani et al., 2013). Due to the population specificity of these haplotypes, it is believed that the sickle cell mutation arose independently in populations and remained to this day (Kulozik et al., 1986). Advances in genomic technologies and data sciences are enabling more targeted disease diagnostics and treatment approaches that can inform health and disease management at the individual level (Mulder et al.., 2018). Currently with the increasing standards of living in many Africa countries, a number of individuals can afford to meet the costs of available SCD management options. Therefore, there is an urgent need for African institutions to train genetic counselors who must be equipped with the current knowledge of available SCD management options. Currently, the use of genomics in the clinical setting in Africa is dominated by the genotyping of genes with the aim of elucidating genetic etiologies for disorders such as SCD and Downs syndrome (Kromberg et al., 2013; Mitropoulos et al., 2017) but this service is still largely unavailable in many healthcare settings in Africa (Munung et al., 2018). Furthermore, complex diseases are becoming more prevalent in Africa, due to environmental risk factors that are associated with increasing urban and more sedentary lifestyles (Tishkoff & Williams, 2002).

Genomic educational efforts should extend hemoglobinopathies and glucose‐6‐phosphate dehydrogenase deficiency since there is documented evidence of more‐robust genetic underpinning for high burden of mental and neurological disorders in Africa (Quansah & McGregor, 2018). The management of the above disorders requires both trained professionals who can offer both psychosocial and genetic counseling to affected individuals/families in communities.

Infant mortality can fall below the local birth‐rate of severe congenital disorders only if these vulnerable children are appropriately diagnosed and treated early, or if their birth‐rate is reduced; that is, through our proposed multidisciplinary strategies. The hemoglobinopathies (the thalassaemias and SCD) are the most common of the lethal monogenic disorders. They can be managed with reasonable success, and prevented by heterozygote diagnosis and genetic counseling alongside pre‐marital genetic screening and selective abortion of affected pregnancies where applicable (Organization, 1985).

### 3.6 Way forward

Genomics literacy and education in Africa is generally still low however some institutions are reviewing their medical curricula to address this challenge therefore bolstering a key step in developing literacy tools in Africa and promoting networks for collaboration. This activity has been majorly spearheaded by the H3Africa consortium (Mulder et al., 2018) and a number of other partnerships between African institutions and the Western countries.

**Figure 2:**
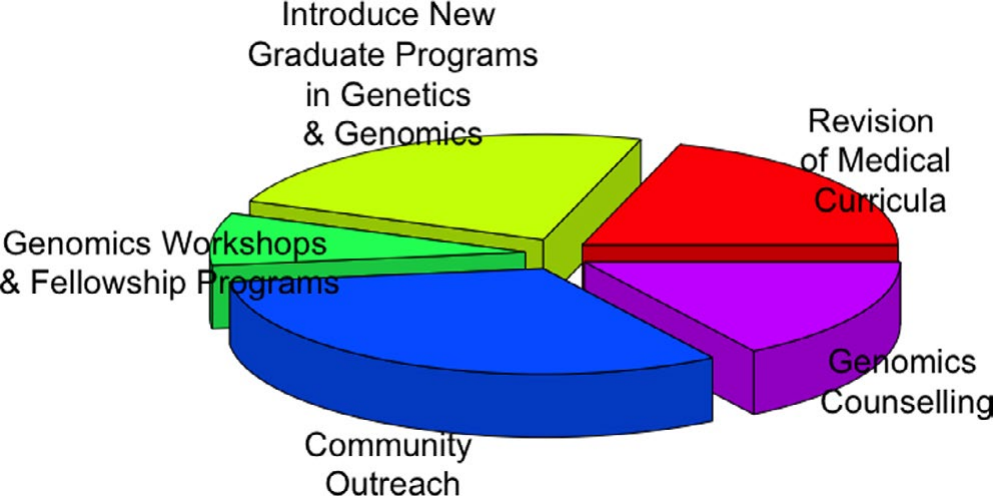
Genomics literacy and awareness initiatives to reduce the burden of common genetic diseases in Africa

Recent progress in understanding human hereditary diseases and in developing approaches that can be applied at the community level, has led to the differentiation of a group of community genetics services, relevant to many aspects of primary health care. Our goal is to identify and describe opportunities that can be adopted at different levels to reduce genetic diseases through genomics‐related educational initiatives that advance genomic literacy in Africa. In addition, we summarize our recommendations in Figure 2 above. In this era of genomic medicine that promises to change the way healthcare is delivered, the African continent presents a unique environment characterized by the highest multilingual and multicultural setting and therefore requires a multidisciplinary approach to increase genomic literacy.

In order for the translation of genomic information to improve medicine and public health, individuals will need to be able to understand the information provided to them and use that information to make health decisions and participate in public policy discussions about genomics (Green, Guyer, & Institute, 2011; Hurle et al., 2013; Lea, Kaphingst, Bowen, Lipkus, & Hadley, 2011). Genomic information in the form of family health history is also critical to improving individuals’ health (Guttmacher, Collins, & Carmona, 2004). This would produce a society that is knowledeagble about the utility of genomics in health and disease leading to a reduction in the incidence of genetic diseases such as SCD.

Countries in Africa can choose to embrace the above proposed methods depending on their national priorities and financial capacity. However, generally the number one hindrance to implementing the above approaches are both “bad” politics and governance, these probably remain the number one risk factor for health improvement in Africa.

## 4 CONCLUSIONS

Integrating genetic and genomic information into all facets of healthcare practice and society in Africa has great potential in reducing the burden of genetic diseases and requires a multidisciplinary strategy that can be adopted in whole or part by the different nations or regions within their strategic plans. Increased genomic literacy among both health care professionals and the general public will see improved better acceptance of both the promises of "genomic era" and "personalized medicine" in Africa, which would ultimately translate into improved health (Dressler, Jones, Markey, Byerly, & Roberts, 2014).

## CONFLICT OF INTERESTS

The authors declare that they have no conflict of interests.
